# Activated Carbon from *Spartina alterniflora* and Its N-Doped Material for Li-Ion Battery Anode

**DOI:** 10.3390/nano15090658

**Published:** 2025-04-26

**Authors:** Hong Shang, Xinmeng Hao, Yougui Zhou, Jia Peng, Lihua Guo, Huipeng Li, Bing Sun

**Affiliations:** School of Science, China University of Geosciences (Beijing), Beijing 100083, China

**Keywords:** *Spartina alterniflora*, activated carbon, N-doped, anode material, lithium-ion battery

## Abstract

The rampant growth of *Spartina alterniflora* has been wreaking havoc on the coastal ecosystems, leading to a serious environmental challenge in recent years. One potential solution to this issue involves converting *Spartina alterniflora* into activated carbon, offering a potential remedy for pollution while creating value in energy storage applications. Herein, through a facile carbonization process with sodium hydroxide activation, we successfully transformed obsolete *Spartina alterniflora* into a porous carbon material (called SAC) and its nitrogen-doped derivative (denoted as SANC) by using melamine as the nitrogen source in a similar procedure. The amorphous structure of these materials was confirmed to enhance lithium-ion storage and electrolyte permeation, making them ideal for use as anodes in lithium-ion batteries. As a result, both SAC and SANC, derived from *Spartina alterniflora*, exhibited outstanding electrochemical performance including high capacity (456.7 and 780.8 mA h g^−1^ for SAC and SANC, respectively, at the current density of 6 mA g^−1^), excellent rate performance (from 6 to 600 mA g^−1^) and long-term cycling stability. Notably, compared to SAC, its N-doped derivative SANC showed superior properties in the battery (retaining a reversible capacity of 412.9 mA h g^−1^ at the current density of 6 mA g^−1^ even after 600 repeated charge–discharge cycles), demonstrating the significantly positive impact of heteroatom doping. This work not only offers a strategy to mitigate environmental challenges but also demonstrates the potential for converting waste biomass into a valuable resource for energy storage applications.

## 1. Introduction

As one of the advanced electrochemical energy storage devices, lithium-ion (Li-ion) batteries have been widely used in modern applications including smart mobile machines, electric devices and vehicles [1,2]. Traditionally, graphite was most applied as anode material due to its excellent conductivity and perfect cycling stability, with a theoretical capacity of 372 mA h g^−1^. Typically, graphite is a crucial and expensive raw carbon either extracted or artificially manufactured from petroleum coke raw materials [3]. Therefore, it is imperative to enhance new electrode materials to promote the energy storage performance of Li-ion batteries and meet the increasing energy demands.

Nowadays, biomass has been receiving increasing attention in energy storage and conversion due to its advantages such as affordability, accessibility, rapid regeneration and eco-friendliness [4,5,6]. Carbon materials derived from biomass usually exhibit micro-nanostructures, large specific surface areas and structural stability, which can facilitate the reversible storage of Li-ion and shorten their transport pathways [7,8,9]. Moreover, heteroatom doping (e.g., B, N, P and S) can regulate the active sites for enhancing the interfacial reactivity and conductivity of derived carbon materials, thereby improving the electrochemical performance [10,11,12,13]. Various biomass sources have been used as precursors for carbonaceous materials and their derivatives, serving as anodes in Li-ion batteries (Table S1) [14,15]. Xiong et al. [16] explored S and N co-doped carbon using waste cotton balls through a simple pyrolysis method and used it as anode material in high-performance Li-ion battery. By a carbonization procedure, Sopon Butcha et al. [17] converted harmful floating plant water hyacinth to useful anode material with high specific capacity and perfect rate performance. Agricultural residues like corn straw [18] and sugarcane bagasse [19] have also been used as high specific capacitance electrodes and showed great potential in energy storage applications. In recent years, the widespread reproduction of Spartina alterniflora brought significant environmental pollution, serious ecological impacts and economic losses. Due to its abundant cellulose, lignin and pore structure [20,21], Spartina alterniflora can benefit from the storage of Li-ion and should be a potential anode material in Li-ion batteries.

**Scheme 1:**
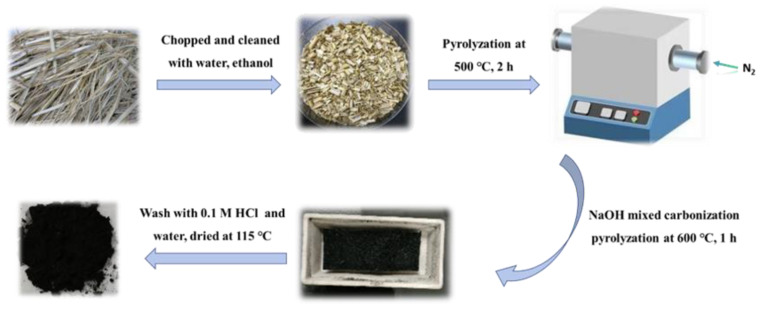
The schematic diagram of the preparation of SAC originated from *Spartina alterniflora*.

In this paper, a porous carbon material (denoted as SAC) was successfully prepared from the obsolete *Spartina alterniflora* through a pyrolyzation method with a straightforward activation process using sodium hydroxide (as shown in Scheme 1). After post-doping with melamine as the nitrogen source, its N-doped activated derivative (denoted as SANC) was also obtained (Scheme S1). Various characterization techniques were employed to characterize the composition, porous structure and morphology of these carbon materials, including X-ray diffraction (XRD), Raman spectroscopy, Brunauer–Emmett–Teller (BET) measurements, X-ray photoelectron spectroscopy (XPS), scanning electron microscope (SEM), transmission electron microscope (TEM) and energy dispersive spectroscopy (EDS). As the anode materials for Li-ion batteries, SAC and SANC originated from *Spartina alterniflora* showed excellent electrochemical performance including high capacity (456.7 and 780.8 mA h g^−1^ for SAC and SANC, respectively, at 6 mA g^−1^), good rate performance (from 6 to 600 mA g^−1^), as well as good cycling stability (265.6 and 412.9 mA h g^−1^ after 600 cycles for SAC and SANC, respectively). Compared to SAC, its N-doped activated derivative SANC showed more perfect properties in Li-ion battery, demonstrating the important impact of heteroatom doping. This work provides an approach to retard the environmental challenges that originate from wreaking havoc on invasive species. More importantly, it can make the waste into a profitable resource and benefit the field of energy storage.

## 2. Experimental

### 2.1. Material Synthesis

The *Spartina alterniflora* was reaped from the east coast of China. After thoroughly washed with distilled water and ethanol, the chopped *Spartina alterniflora* was grinded into powder using a high-speed multifunctional grinder. Then, the *Spartina alterniflora* powder was pre-carbonized in a tube furnace under an argon atmosphere. After the pre-carbonization, the product was mixed with NaOH in a 1:1 mass ratio and thoroughly ground with a mortar. 600 °C was applied to activate the material and then HCl (0.1 mol L^−1^) was used to regulate the Ph until neutral. After the filtration and drying process, SAC material was successfully obtained. To prepare the N-doped activated derivative SANC material, melamine was used as the nitrogen source. It was first mixed with *Spartina alterniflora* powder in a 1:1 mass ratio during the pre-carbonization process. The subsequent treatment steps followed the similar prepared procedure for SAC.

### 2.2. Characterization

XRD spectra were measured by Empyrean diffractometer (Cu Kα radiation, with an output power of 1.6 kW at a voltage of 40 kV) (Malvern Panalytical, Malvern, UK). Renishaw-2000 Raman spectrometer was used to measure Raman spectra with an Ar laser at 473 nm (Wotton-under-Edge, UK). Thermo Scientific ESCALab 250Xi apparatus using 200W mono-chromated Al Kα radiation (Boston, MA, USA) was used to measure XPS spectra. BET (ASAP2020) (Belsorp-max, Osaka, Japan) N_2_ adsorption–desorption tests were carried out at 77 K to measure the specific surface area and pore information of the materials. SEM images, elemental distributions and TEM images were measured by Hitachi Model S-4800 field emission scanning electron microscope and HT-7700 electron microscope (an accelerating voltage of 100 kV) (Ibaraki, Japan).

### 2.3. Electrochemical Testing

The prepared SAC and SANC materials (90%) were fully mixed with carbon black (5%) and polyvinylidene fluoride (PVDF, 5%) with the N-methyl-2-pyrrolidinone (NMP) into a slurry mixture. After placed on the Cu foil, the slurry mixture was dried in a vacuum oven. Before the fabrication, the foil was slowly cut into small pieces with a diameter of 12 mm. Then, the CR2032 button batteries were assembled in an argon-filled glove box (in which the O_2_/H_2_O content was below 0.01 ppm), with opposite lithium metal electrode, Celgard 2400 PP (Celgard, Charlotte, NC, USA) polypropylene separator and EC/DMC = 1:1 liquid electrolyte with 1.0 M LiPF_6_. The average mass of loading active SAC/SANC was 1 mg cm^−2^. For the battery electrochemical performance, the CT2001A (Wuhan, China) land battery system was used in the voltage range from 5 mV to 3 V. The cyclic voltammograms and the electrochemical impedance spectroscopy were measured on CHI760E (Shanghai, China) electrochemical workstation (scan rate of 0.5 mV s^−1^, frequency range 0.01 Hz to 100 kHz).

## 3. Results and Discussion

**Figure 1:**
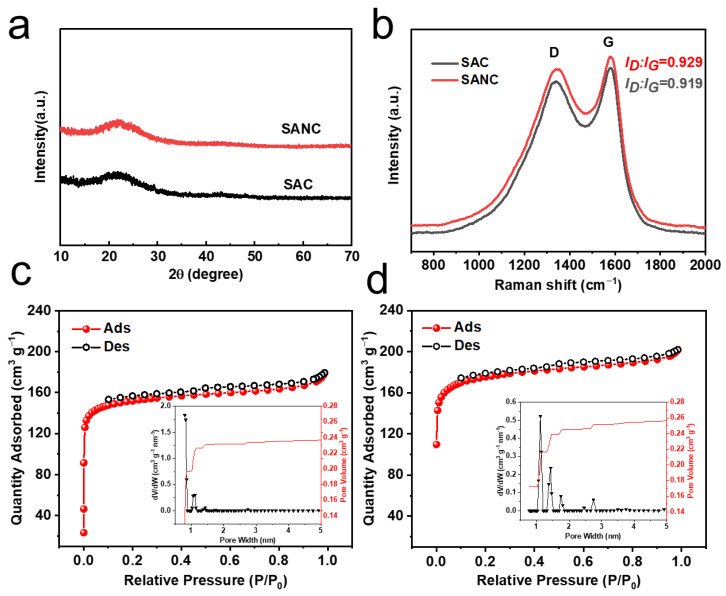
(**a**) XRD patterns; (**b**) Raman spectra; (**c**,**d**) N_2_ adsorption–desorption isotherms of SAC and SANC (inset: pore size distribution and pore volume).

The formation of carbonaceous derivatives from *Spartina alterniflora* was confirmed by structural characterizations using XRD, Raman, BET and XPS. The XRD patterns of SAC and SANC in Figure 1a showed a peak at around 22° ((002) plane of graphite carbon) [22]. The broad diffraction patterns with lower intensity also demonstrated their amorphous nature and limited graphitization. The partial graphitization of carbon may benefit the electrochemical performance as an anode by effectively increasing the reversible capacity of Li-ion intercalation [23]. Increasing the spacing between the layers of carbon material can enhance Li-ion transfer efficiency, thereby improving the cycling and rate performance of Li-ion batteries. Notably, the (002) diffraction peak of the N-doped SANC material (22.4°) exhibited a higher 2θ value compared to SAC (21.8°). This was due to the active nitrogen atoms, which can efficiently promote the transportation of lithium within the pores and increase the disordered property of the biomass structure [24]. Raman spectroscopy is a useful technology to evaluate the graphitization degree [25]. As shown in Figure 1b, these two carbon materials showed similar characteristic peaks at 1380 cm^−1^ (D band, representing the defects of graphitic carbon) and 1595 cm^−1^ (G band, representing the degree of graphitization). The intensity ratio (*I_D_*/*I_G_*) is usually utilized to demonstrate the degree of amorphous structure in the prepared carbon material [26,27]. As a result, the *I_D_*/*I_G_* values were calculated as 0.919 for SAC and 0.929 for SANC, respectively, indicating that nitrogen-doping introduced more structural defects and increased the amorphous nature. Compared to the ordered carbon material, the defects, and/or disordered structure are beneficial for enhancing the active sites, thus potentially resulting in the improved electrochemical performance when employed as the anodes in Li-ion batteries.

To detect the specific surface areas and pore size distributions of SAC and SANC, N_2_ adsorption/desorption tests were measured. As shown in Figure 1c,d, the two carbon materials exhibited a characteristic type *I* adsorption–desorption isotherms and a distinct hysteresis loop at relative high pressure, demonstrating their microporous structures of the biomass carbons from *Spartina alterniflora*. The corresponding specific surface areas were calculated to be 569.148 m^2^ g^−1^ for SAC and 657.897 m^2^ g^−1^ for SANC, respectively. The pore size distribution curves in Figure 1c,d were used to confirm the microporous features. The microporous diameters mainly distributed between 1 and 3 nm, which is beneficial to the enhanced Li-ion storage and facilitates the permeation of electrolyte [28]. Based on the BET results, it can be predicted that the N-doped derivative SANC may have more superior performance as anode electrodes due to its higher specific surface area and abundant active defects, which can increase the storage of lithium.

**Figure 2:**
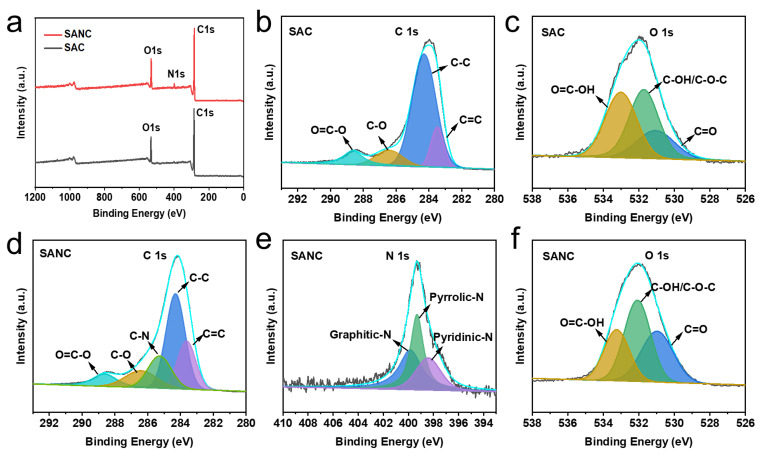
(**a**) XPS spectra; (**b**,**c**) high-resolution C 1 s and O 1 s spectra of SAC; (**d**–**f**) high-resolution C 1 s, N1s and O 1 s spectra of SANC.

The surface chemical composition of the carbon materials SAC and SANC were realized by XPS measurements. As can be seen in Figure 2a, in the XPS survey spectrum of SAC, C (85.01%) and O (14.1%) can be observed. In SANC, the contents were 80.82% for C (284.4 eV), 4.22% for N (400.6 eV) and 14.95% for O (533.2 eV), respectively. This phenomenon indicated that N was successfully doped into the activated carbon SAC. Figure 2b–f and Table 1 displayed the high-resolution spectrum of C1s, N1s and O1s, respectively. As can be seen in the C1s spectrum of SAC in Figure 2b, there were four peaks at 283.62 (C−C bond), 284.29 (C=C bond), 286.41 (C−O bond) and 288.62 eV (O=C−OH bond), respectively [29]. In the high-resolution O1s spectrum in Figure 2c, three peaks at 531.06 (C=O bond), 532.08 (C−OH/C−O−C bond) and 533.22 eV (O=C−OH bond) can be observed [30]. For the N-doped activated derivative SANC material, the C1s spectrum can be deconvoluted into five peaks at 284.29 eV (C−C bond), 283.62 eV (C=C bond), 285.27 eV (C−N bond), 286.41 eV (C−O bond) and 288.62 eV (C=O bond), respectively. There were three peaks in the high-resolution N1s spectrum at 398.42 (pyridinic nitrogen), 399.29 (pyrrolic nitrogen) and 399.83 eV (graphitic nitrogen), respectively [31]. In the high-resolution O1s spectrum, there were three characteristic peaks at 531.06 (C=O bond), 532.08 (C−OH/C−O−C bond) and 533.22 eV (O=C−OH bond), respectively. According to the reported literature [32,33], the doping of nitrogen can promote the formation of functional groups and increase active defects for Li-ion storage, thus benefiting to the enhancement of the electrochemical performance.

**Figure 3:**
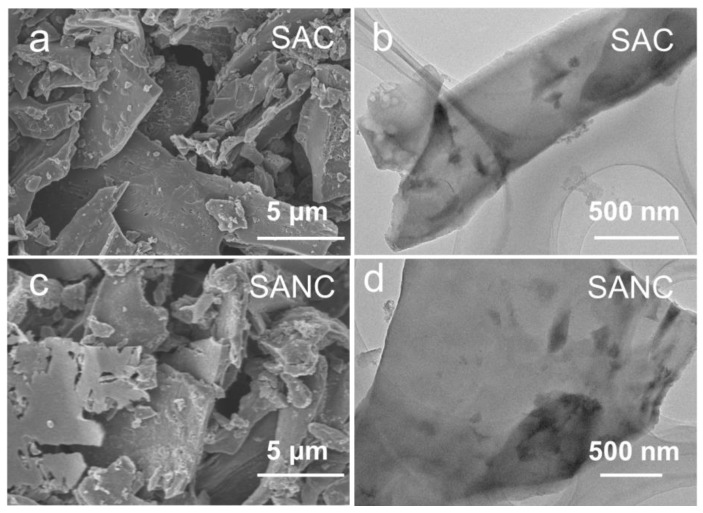
SEM images and TEM images of SAC (**a**,**b**) and SANC (**c**,**d**).

Figure 3, Figures S1 and S2 showed the morphologies of the two carbon materials derived from *Spartina alterniflora*, including SEM, TEM and EDS. The activated carbon materials SAC and its N-doped derivative SANC all present irregular carbon blocks (Figure 3a,c), demonstrating their amorphous nature, consistent with the XRD results. As shown in SEM images, there were many pore structures in the bulk carbons, which also can be confirmed by the BET results. The TEM images of SAC and SANC were exhibited in Figure 3b,d. It was obvious that there were no visible lattice fringes, which was attributed to their limited graphitization degree as well as the amorphous characteristics. Compared to the SAC, after the successful N-doping by melamine, the N was uniformly distributed in SANC (Figure S1). Heteroatom doping in carbon materials is an efficient approach to induce plenty of active sites and defects [34,35]. Consequently, the N-doping would have a positive effect to regulate the electronic and chemical structure and enhance the electrochemical reactivity and performance.

**Figure 4:**
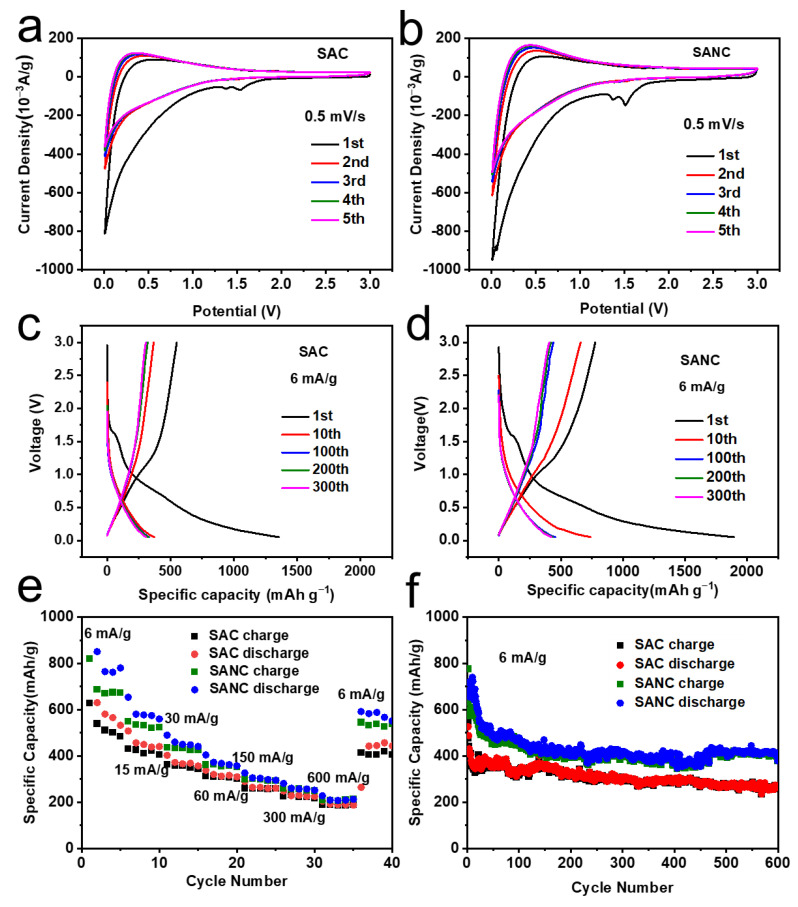
(**a**) Cyclic voltammetry curves of SAC electrode (0.5 mV·s^−1^); (**b**) cyclic voltammetry curves of SANC electrode (0.5 mV·s^−1^); (**c**,**d**) voltage-capacity curves of SAC and SANC electrodes at 6 mA g^−1^; (**e**) rate performance of SAC and SANC electrodes at different current densities; (**f**) cycling performance of SAC and SANC electrodes (1C = 300 mA g^−1^).

To evaluate their potential in energy storage, the two materials SAC and SANC from *Spartina alterniflora* were used as the lithium storage material in the Li-ion battery. The electrochemical properties of SAC and SANC were first measured by cyclic voltammetry (CV) (0.01-3.0 V, 0.5 mV s^−1^). Figure 4a showed the CV of the SAC electrode in the initial five cycles. Firstly, there were two small cathode peaks, which disappeared in the subsequent cycles. This phenomenon can be attributed to some irreversible reactions related to the electrolyte decomposition on the surface of the electrode and the formation of the solid electrolyte interphase (SEI) film [36,37]. This is also the main reason for the low Coulomb efficiency (44%) in the initial process. In the following measurements, CV curves almost overlapped, indicating the good structural stability of the electrode. For the SANC electrode, the CV just presented similar curves compared to the SAC material, indicating that there was no significant difference between the structure of SAC and its N-doped derivative SANC.

Figure 4c,d showed the constant charge–discharge processes of the two materials at a current density of 6 mA g^−1^ from 0.01 to 3 V. The first charge specific capacity of the SANC sample (Figure 4d) can reach 776.3 mA h g^−1^, and the initial Coulomb efficiency is 41%, lower than that of SAC. During the first cycle near 1.6 V, there is a small platform, which was attributed to the formation of SEI and the degradation of the electrolyte [38]. The specific capacity can maintain 399.5 mA h g^−1^ even after 300 repeated charge–discharge cycles, which was larger than the carbon material SAC (290.5 mA h g^−1^) before N-doping. Rate capabilities of the two electrodes were evaluated at different current densities ranging from 6 to 600 mA g^−1^. In Figure 4e, the N-doped SANC electrode showed reversible capacities of 780.8, 580.6, 459.7, 372.5, 305.2, 260.1 and 212.3 mA h g^−1^ at 6, 15, 30, 60, 150, 300 and 600 mA g^−1^, respectively, higher than that of SAC electrode (456.7, 421.2, 326.5, 273.5, 229.9, 158.3 and 117.8 mA h g^−1^ at the same current densities), demonstrating more excellent Li-ion storage ability and rate performance of SANC than SAC. When the current density returned to 6 mA g^−1^, 445.0 and 588.4 mA h g^−1^ were obtained for SAC and SANC. After N-doping, the cycling performance was also improved. At the current density of 6 mA g^−1^ in Figure 4f and Figure S3, the SANC electrode exhibited excellent cycling stability. Even after 600 repeated charge–discharge cycles, the battery can retain a capacity of 412.9 mA h g^−1^. In contrast, the SAC electrode achieved a reversible capacity of 265.6 mA h g^−1^ under the same condition, lower than that of SANC electrode. The SANC electrode also showed superior cycle stability with a reversible capacity at a higher current density of 600 mA g^−1^ (Figure S4). Owing to the environment interference and the internal polarization during long-term tests, for the rate performance and the cycle stability, there were slight fluctuations in the capacities.

**Figure 5:**
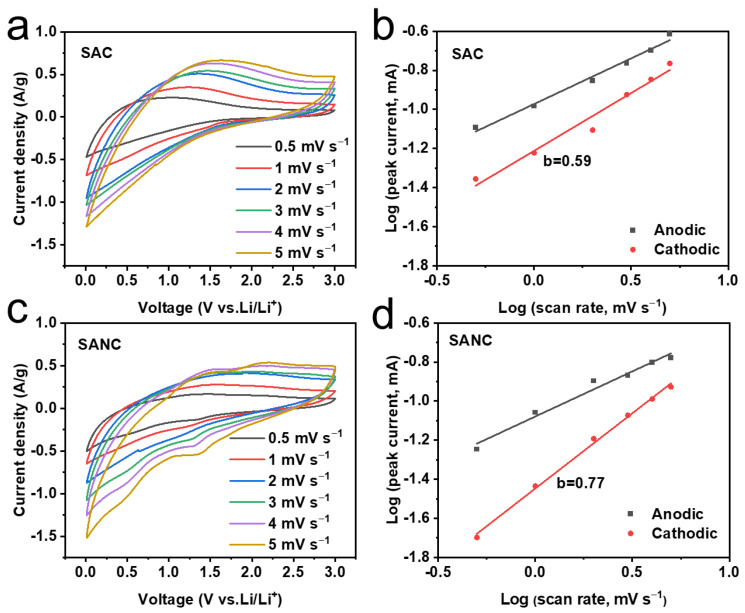
(**a**) CV curves at different scanning rates for SAC; (**b**) determination of the b-value using the relationship between peak current and scan rate for SAC; (**c**) CV curves at different scanning rates for SANC; (**d**) determination of the b-value using the relationship between peak current and scan rate for SANC.

To determine the Li-ion storage processes of the two materials and the electrochemical kinetic behaviors in Li-ion batteries, CV curves at different scanning rates (*v*) ranging from 0.5 to 5 mV s^−1^ were measured for SAC and SANC (Figure 5). As shown in Figure 5a,c, with the increase in scanning speed, the current density enhanced and the peak potential shifted, demonstrating that the intercalation and de-intercalation processes of lithium in SAC and SANC were reversible. According to the relationship between the measured current (*I*) and the scanning rate (*v*), *I* = *av^b^* [39], the b value of the SAC electrode was 0.59, more approximately to 0.5. This indicated that the electrochemical reaction of SAC anode in the Li-ion battery was mainly a diffusion-controlled process. Diffusion control implies a slower Li-ion transmission rate, which becomes the limiting step in the electrochemical reaction. When the *b* value is closer to 1, it suggests a mainly capacitance-controlled electrochemical reaction. Capacitance control indicates a faster charge transfer rate on the electrode surface, which is the primary factor influencing the electrochemical reaction rate. As a result, after N-doping, the faster surface charge transfer rate of the SANC electrode (with b value of 0.77), along with rapid adsorption and desorption processes, contributed to the improved rate performance.

To further explore the impact of N-doping in SAC on the electrochemical process of Li-ion storage, the electrochemical impedance spectroscopy (EIS) was investigated. As shown in Figure S5, the initial electrochemical impedance spectra of SAC and SANC showed that in the high-frequency region, the Nyquist plots of SAC and SANC electrodes exhibited semi-circular shapes, while in the low-frequency region, they all displayed sloping lines. In Figure S6, after 100 charge–discharge repeated cycles, the interface resistance was almost the same to the initial electrochemical impedance, indicating that during the charging and discharging process, a stable SEI film had been formed, which could maintain the structure of the material. In general, the semi-circle in the high-frequency region represents the charge transfer resistance, while the sloping line in the low-frequency region represents the impedance related to ion diffusion processes in anode material [40,41]. Compared to the SAC electrode, the diameter of the semi-circle in the SANC electrode was reduced, indicating that after N-doping into the SAC material, a decreased charge transfer resistance at the electrode/electrolyte interface was obtained, which can contribute to the improved electrochemical performance of the SANC.

## 4. Conclusions

In conclusion, activated carbon derived from *Spartina alterniflora* as well as its N-doped material were prepared using a simple strategy. Benefiting from their porous structures, the storage of lithium and the permeation of electrolytes would be promoted and the diffusion distance of lithium ions would be reduced. Especially, in the N-doped material, more defects were produced and can be considered as active sites. Consequently, compared to the activated carbon from *Spartina alterniflora*, its N-doped carbon derivative showed superior electrochemical performance when utilized as anode material of Li-ion battery. Even after 600 repeated charge-discharge cycles, the N-doped material SANC can still provide a reversible high capacity of 412.9 mA h g^−1^. The exceptional electrochemical performance of the two materials in Li-ion batteries presented here offers a cost-effective and simple solution to address the encroachment issue posed by *Spartina alterniflora* species and can effectively turn waste into a valuable resource and benefit energy storage and conversion.

## Figures and Tables

**Table 1:** XPS C1s analysis of the elemental composition of SANC and SAC.

**Sample**	**Peaks and Their Positions (eV)**				
	**C=C**	**C−C**	**C−N**	**C−O**	**O=C−OH**
	283.62	284.29	285.27	286.41	288.62
SAC	18.1%	45.0%	14.9%	12.8%	9.2%
SANC	17.6%	57.9%		13.14%	11.34%

## Data Availability

The datasets used and/or analyzed during the current study are available from the corresponding author on reasonable request.

## Supplementary Materials

The following supporting information can be downloaded at: https://www.mdpi.com/article/10.3390/nano15090658/s1. Scheme S1. The schematic diagram of the synthesis procedure of SANC. Figure S1. Elemental distribution of SANC material. Figure S2. Elemental distribution of SAC material. Figure S3. Coulombic efficiency of SAC and of SANC electrodes. Figure S4. Cycling performance of SAC and SANC electrode at 600 mA g^−1^. Figure S5. The initial electrochemical impedance spectra and corresponding equivalent circuit of SAC and SANC. Figure S6. Electrochemical impedance spectra of SAC and SANC after 100 charge-discharge repeated cycles. Table S1. Performance comparison of biochar carbon as anodes in LIBs [42,43,44,45,46].
