# Supplementary material for: Activated Carbon from Spartina alterniflora and Its N-Doped Material for Li-Ion Battery Anode
Source: Nanomaterials (Basel). 2025 Apr 26;15(9):658. doi: 10.3390/nano15090658 (PMC12073150; doi:10.3390/nano15090658)
Supplement: Supplementary file 1 [file nanomaterials-15-00658-s001.zip › nanomaterials-3601704-supplementary.pdf]

Activated carbon from *Spartina alterniflora* and its N-doped material for Li-ion battery anode

Hong Shang [1,*], Xinmeng Hao [1], Yougui Zhou [1], Jia Peng [1], Lihua Guo [1], Huipeng Li [1] and Bing Sun [1,*]

[1] School of Science, China University of Geosciences (Beijing), Beijing, 100083, P. R. China
* Correspondence: shanghong@cugb.edu.cn (H.S.); sunbing@cugb.edu.cn (B.S.)

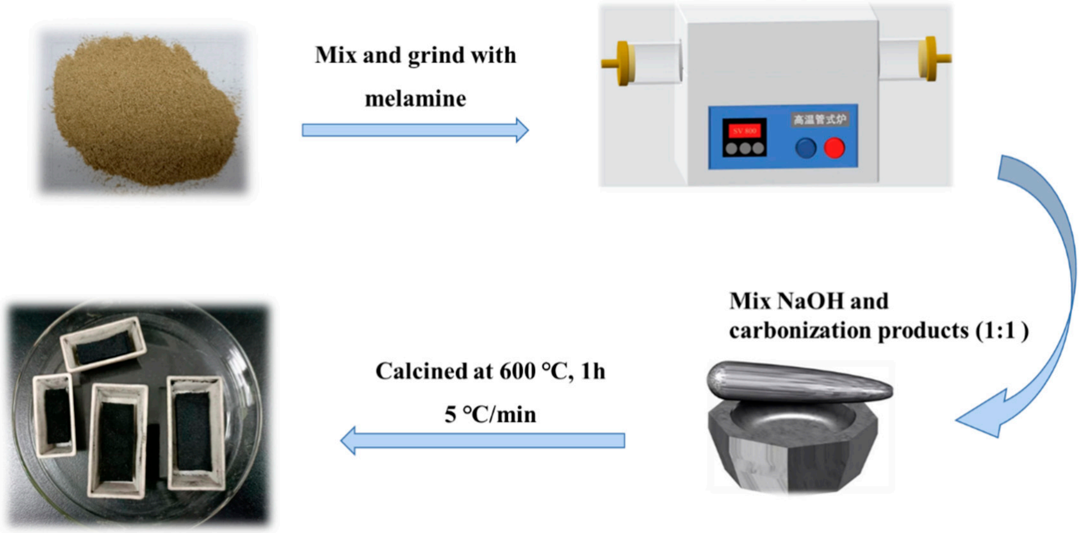

Scheme S1. The schematic diagram of the synthesis procedure of SANC.

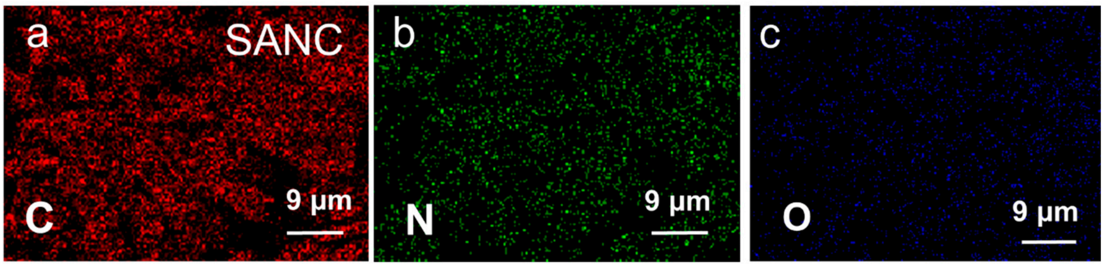

Figure S1. Elemental distribution of SANC material.

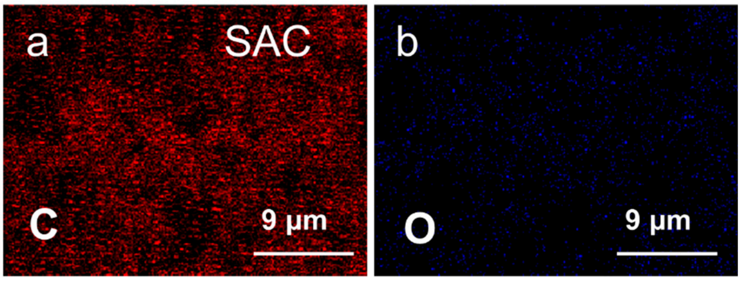

Figure S2. Elemental distribution of SAC material.

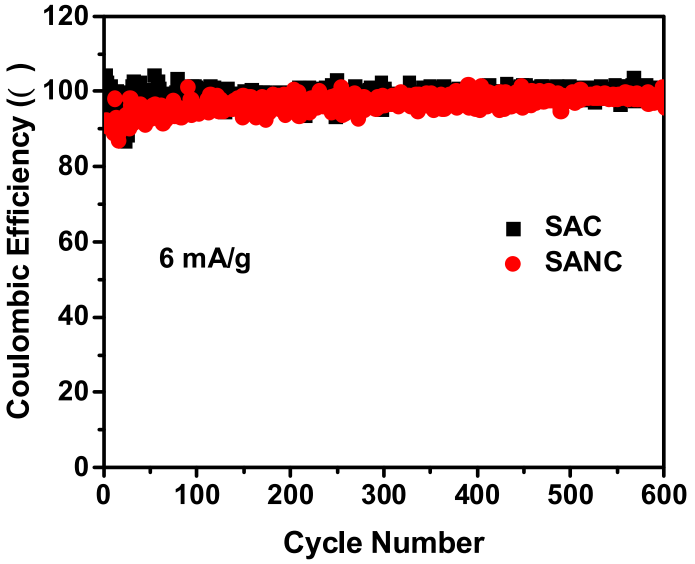

Figure S3. Coulombic efficiency of SAC and of SANC electrodes.

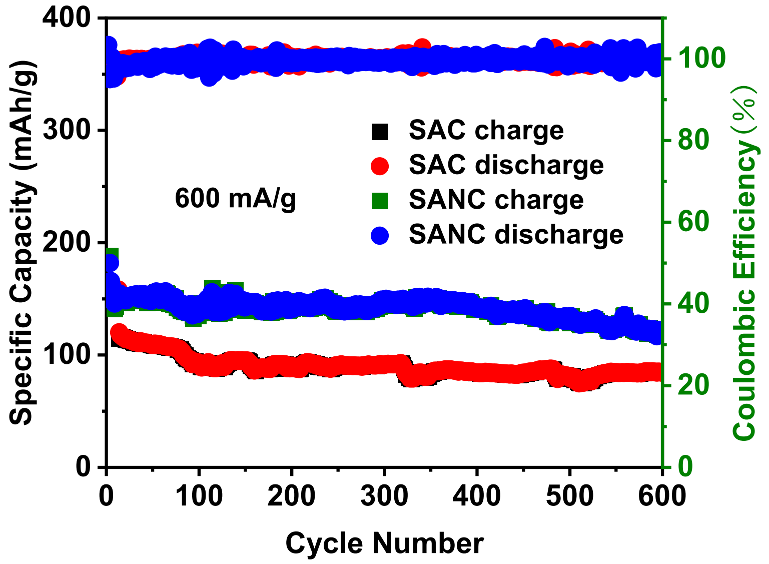

Figure S4. Cycling performance of SAC and SANC electrode at 600 mA g^{-1}.

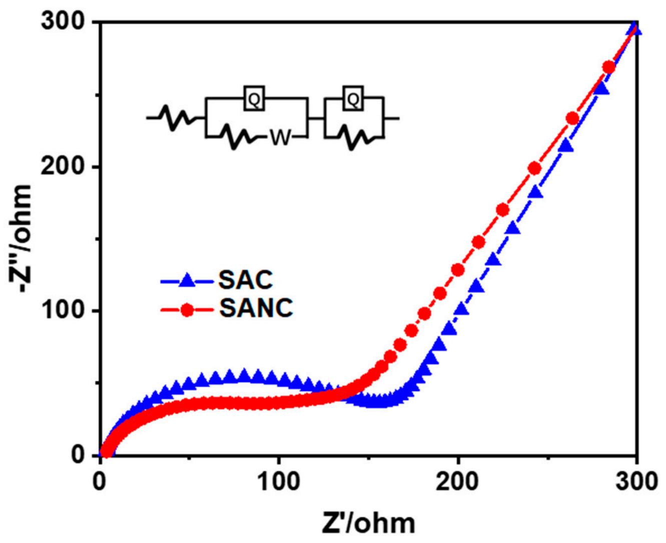

Figure S5. The initial electrochemical impedance spectra and corresponding equivalent circuit of SAC and SANC.

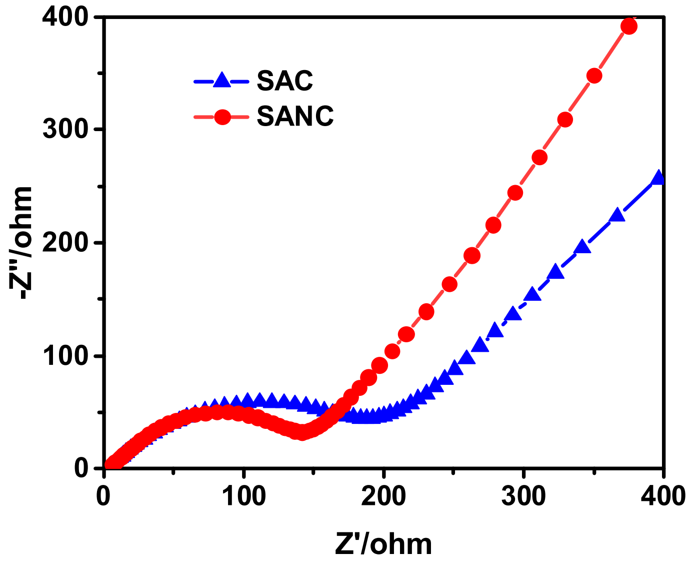

Figure S6. Electrochemical impedance spectra of SAC and SANC after 100 charge-discharge repeated cycles.

Table S1. Performance comparison of biochar carbon as anodes in LIBs.

Biomass feedstock	Functionality doping	Specific capacity (mAh g^{-1})	Current density (mA g^{-1})	Ref.
SAC		372.5	60	this work
SANC	N	273.5	60	this work
Rice Straw	Si	352	37.2	[42]
Sawdust	Ni, Mn, Fe	293	20	[43]
Ahnfeltia tobuchiensis	N	293	36	[44]
Cotton stalk		271.7	100	[45]
Corncob	N	232.1	100	[46]
